# Supplementary material for: Single-cell nascent RNA sequencing unveils coordinated global transcription
Source: Nature. 2024 Jun 5;631(8019):216–23. doi: 10.1038/s41586-024-07517-7 (PMC11222150; doi:10.1038/s41586-024-07517-7)
Supplement: Supplementary file 1 — This file contains descriptions for Supplementary Tables 1–6 (Tables supplied separately), Supplementary Tables 7–9 and Supplementary Figs 1–4. [file 41586_2024_7517_MOESM1_ESM.pdf]

nature portfolio

https://doi.org/10.1038/s41586-024-07517-7

Supplementary information

Single-cell nascent RNA sequencing unveils coordinated global transcription

In the format provided by the authors and unedited

nature portfolio

https://doi.org/10.1038/s41586-024-07517-7

Supplementary information

Single-cell nascent RNA sequencing unveils coordinated global transcription

In the format provided by the
authors and unedited

Single-cell nascent RNA sequencing unveils coordinated global transcription

Dig B. Mahat[1], Nathaniel D. Tippens[1], Jorge D. Martin-Rufino[2], Sean K. Waterton[1,3], Jiayu Fu[1,4], Sarah E. Blatt[1,5], Phillip A. Sharp[1,6]

[1]Koch Institute for Integrative Cancer Research and Department of Biology, Massachusetts Institute of Technology, Cambridge, MA 02139
[2]Broad Institute of MIT and Harvard, Cambridge, MA 02142
[3]Current address: Department of Biology, Stanford University, Stanford, CA 94305
[4]Current address: Interdisciplinary Biological Sciences Graduate Program, Northwestern University, Evanston, IL 60208
[5]Current address: Exact Sciences Corporation, Madison, WI 53719
[6]Lead Contact

Table of contents:

Supplementary Figure 1 | Uncropped polyacrylamide gel electrophoresis blots from Extended Data Figure 1. a, Polyacrylamide gel electrophoresis blot for **Extended Data Figure 1c** showing the incorporation efficiency of 3'-(O-Propargyl)-ATP or 3'-Azido-3'-dATP by native RNA polymerase in nuclear run-on reaction. The propargyl or azide labeled nascent RNA is clicked with Cy5 via CuAAC (Azide-Cy5 or Alkyne-Cy5) or SPAAC (DBCO-Cy5), resolved in a denaturing polyacrylamide gel electrophoresis and quantified by measuring the Cy5 fluorescent from the gel image. **b,** Polyacrylamide gel electrophoresis blots for **Extended Data Figure 1d** showing the relative quantification of reverse transcription (RT) efficiency of two commercial enzymes traversing through the triazole link formed between the alkyne-labeled RNA and azide-labeled DNA by CuAAC. RT was performed in the presence of either native dCTP or radioisotope a-^{32}P dCTP, and the RT reaction was resolved in denaturing polyacrylamide gel electrophoresis and imaged sequentially for nucleic acid signal by staining with SYBR Gold (top gel) and radioisotope signal by ^{32}P Phosphor imaging (bottom gel). **c,** Polyacrylamide gel electrophoresis blot for **Extended Data Figure 1f** showing the confirmation and relative quantification of CuAAC, RT, and PCR of clicked product formed between the alkyne-labeled RNA and azide-labeled DNA by three commercial Reverse transcriptase enzymes, resolved in a denaturing polyacrylamide gel electrophoresis and quantified by staining with SYBR Gold and measuring fluorescence. In panels **a-c**, the purple dotted line represents the gel region shown in Extended Data Figures.

Supplementary Figure 2 | Uncropped polyacrylamide gel electrophoresis blots from Extended Data Figure 3. a, Polyacrylamide gel electrophoresis blot for **Extended Data Figure 3b** showing the relative quantification of nuclear run-on efficiency with various sarkosyl concentrations. Nascent RNA collected after nuclear run-on reaction with either native CTP or click-compatible 3'-(O-Propargyl)-CTP was clicked with Cy5-azide, resolved in denaturing polyacrylamide gel electrophoresis, and imaged for Cy5 fluorescence. **b,** Polyacrylamide gel electrophoresis blot for **Extended Data Figure 3c** shows the effect of different ratios of CuAAC accelerating ligand BTTAA in CuAAC efficiency. RNA-propargyl was clicked with azide-DNA containing Cy5 in the presence of various ratios of BTTAA:$CuSO_4$, resolved in denaturing polyacrylamide gel electrophoresis and imaged for Cy5 fluorescence. **c,** Polyacrylamide gel electrophoresis blot for **Extended Data Figure 3e** shows the effect of the denaturing agent's presence in CuAAC efficiency. The CuAAC reaction was resolved in a denaturing polyacrylamide gel electrophoresis and quantified by staining with SYBR Gold and measuring fluorescence. In panels **a-c**, the purple dotted line represents the gel region shown in Extended Data Figures.

Supplementary Figure 3 | Features of single-cell barcoded DNA adaptors used in scGRO-seq experiments. a, The 86 nt 5'-AzScBc DNA makes a hairpin structure, creating an Eag I restriction site. The 3' end of the adaptor also serves as the extension primer during reverse transcription. **b,** The inclusion of template-switching oligo without CuAAC of nascent RNA on the 43 nt 5'-AzScBc DNA with the ACAGG sequence (top cartoon) forms a substrate sequence for DASH. The CuAAC of nascent RNA fails to generate the substrate for DASH (bottom cartoon).

Supplementary Figure 4 | Equations for the burst size and burst frequency estimation.

Supplementary Table 1. Table_1_Genes_Enhancers_Burst_Kinetics.xlsx
Burst sizes and frequencies of transcribed genes using 5 kb or 10 kb burst windows. Columns are features (gene names contain the “GN-” prefix, and enhancer names contain the “chrXX-” prefix), chromosome, start, end, length of gene or enhancer, number of UMIs per cell per kb, number of reads, number of bursts detected, burst frequency, and burst size.

Supplementary Table 2. Table_2_GSEAresults_Burst_Kinetics.xlsx
Gene Set Enrichment Analysis of burst size and burst frequency. Four tabs represent “GSEA of Burst Frequency” and “GSEA of Burst Size” with columns representing names of gene sets, size of gene sets, enrichment scores, normalized enrichment scores, nominal p-value, FDR q-value, and rank), and “Aff4 Genes” and “MYC Genes” with columns representing symbol, rank in the gene list, rank metric score, running enrichment score, and core enrichment.

Supplementary Table 3. Table_3_CellCycle_genes_Seurat_FindAllFeatures.xlsx
Differentially expressed genes among G1/S, S, and G2/M phases of the cell cycle. Columns are gene name, cell state, average $\log_2$ fold change, pct.1, pct.2, p_value, and adjusted p-value identified by the “FindAllFeatures” function of Seurat (single-cell analysis package).

Supplementary Table 4. Table_4_GxG_cotranscribed_pairs.xlsx
Correlated gene-gene pairs. Columns contain gene-A, gene-B, correlation between gene-A and gene-B, p-value from chi-square test, multiple hypothesis corrected p-value using Benjamini-Hochberg (BH) correction method, the fraction of cells with gene-A transcription, the fraction of cells with gene-B transcription, expected co-transcription of gene-A and gene-B, observed co-transcription of gene-A and gene-B, and empirical false discovery rates of co-transcription of gene-A and gene-B determined from 100, 250, 500, 1,000, 2,500, and 5,000 permutations.

Supplementary Table 5. Table_5_scGROv2p8_GxGmodules_enrichGO_selected.xlsx
Non-redundant Gene Ontology terms and the co-transcribed genes that contribute to the GO terms' enrichment resulted from “enrichGO” function in the clusterProfiler R package. Columns contain ontology class, ontology ID, ontology description, gene ratio, background ratio, p-value, q-value, gene IDs, and counts.

Supplementary Table 6. Table_6_ExG_cotranscription_pairs_within_2500kb.xlsx
Correlated enhancer-gene pairs. Columns contain gene name, enhancer name, the correlation between gene and enhancer, p-value from chi-square test, multiple hypothesis corrected p-value using Benjamini-Hochberg (BH) correction method, strand of the gene, the fraction of cells with gene transcription, the fraction of cells with enhancer transcription, expected co-transcription of enhancer and gene, observed co-

transcription of enhancer and gene, the empirical false discovery rates of enhancer-gene co-transcription determined from 100, 250, 500, 1,000, 2,500, 5,000, and 10,000 permutations, and whether the enhancer-gene pair is co-transcribed or not.

Supplementary Table 7. Single-cell barcoded DNA adaptors used in scGRO-seq experiments. Js represent unique cell barcodes, Ns represent unique molecular index, and SpC3 denotes a three-carbon spacer.

	Sequence	Length	Cell Barcode	UMI
1	5'-[azide-T]JJJJJJJJJJNNNNNNGATCGTCGGACTGTAGAACTCTGAAC-3'	43 nt	10 nt	6 nt
2	5'-[azide-T]NNNNJJJJJJJJJJJJGATCGTCGGACTGTAGAACTCTGCGGCCGTGCTCGTTTTCGAGCACGGCCGCAGAGTTCTACAGTCCGA-3'	86 nt	12 nt	4 nt
3	5'-[azide-T]ACAGGNNNNNJJJJJJJJJJAGATCGGAAGAGCGTCGTGTAG[SpC3]-3'	43 nt	10 nt	5 nt

Supplementary Table 8. Polymerase chain reaction (PCR) program for the amplification of cDNA.

	Steps	Denature (98°C)	Anneal (60°C)	Extension (72°C)	Storage (4°C)
	Initial denaturation	30 sec	-	-	-
6 cycles	Denaturation	10 sec	-	-	-
	Anneal		20 sec	-	-
	Extension			30 sec	
	Final extension	-	-	2 min	-
	Storage	-	-	-	∞

Supplementary Table 9. Polymerase chain reaction (PCR) program for the amplification of DASHed library.

	Steps	Denature (98°C)	Anneal (60°C)	Extension (72°C)	Storage (4°C)
	Initial denaturation	30 sec	-	-	-
6 cycles	Denaturation	10 sec	-	-	-
	Anneal		20 sec	-	-
	Extension			30 sec	
	Final extension	-	-	2 min	-
	Storage	-	-	-	Forever

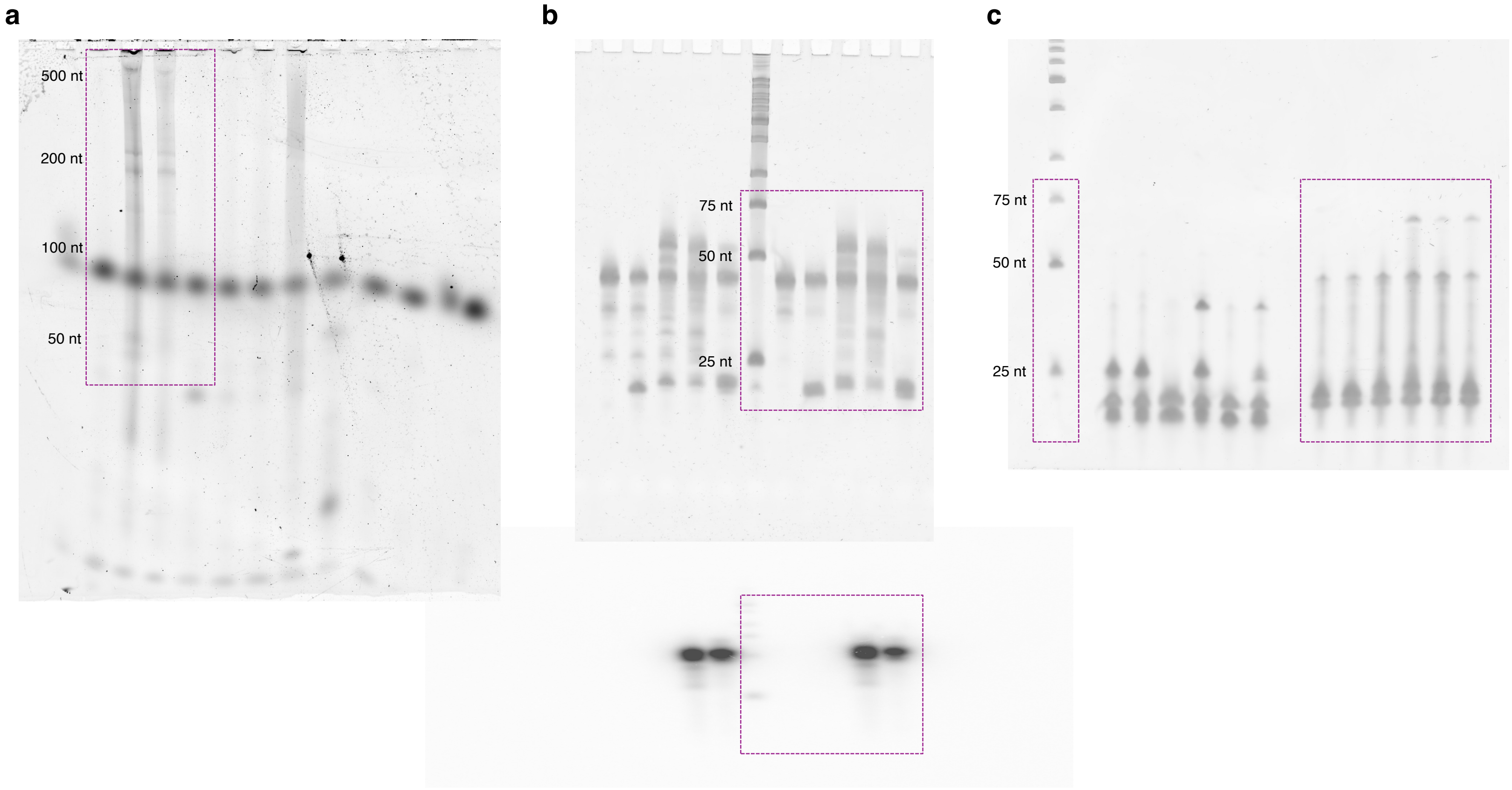

Supplementary Figure 1 | Uncropped polyacrylamide gel electrophoresis blots from Extended Data Figure 1. a, Polyacrylamide gel electrophoresis blot for **Extended Data Figure 1c** showing the incorporation efficiency of 3'-(O-Propargyl)-ATP or 3'-Azido-3'-dATP by native RNA polymerase in nuclear run-on reaction. The propargyl or azide labeled nascent RNA is clicked with Cy5 via CuAAC (Azide-Cy5 or Alkyne-Cy5) or SPAAC (DBCO-Cy5), resolved in a denaturing polyacrylamide gel electrophoresis and quantified by measuring the Cy5 fluorescent from the gel image. **b,** Polyacrylamide gel electrophoresis blots for **Extended Data Figure 1d** showing the relative quantification of reverse transcription (RT) efficiency of two commercial enzymes traversing through the triazole link formed between the alkyne-labeled RNA and azide-labeled DNA by CuAAC. RT was performed in the presence of either native dCTP or radioisotope a-^{32}P dCTP, and the RT reaction was resolved in denaturing polyacrylamide gel electrophoresis and imaged sequentially for nucleic acid signal by staining with SYBR Gold (top gel) and radioisotope signal by ^{32}P Phosphor imaging (bottom gel). **c,** Polyacrylamide gel electrophoresis blot for **Extended Data Figure 1f** showing the confirmation and relative quantification of CuAAC, RT, and PCR of clicked product formed between the alkyne-labeled RNA and azide-labeled DNA by three commercial Reverse transcriptase enzymes, resolved in a denaturing polyacrylamide gel electrophoresis and quantified by staining with SYBR Gold and measuring fluorescence. In panels **a-c**, the purple dotted line represents the gel region shown in Extended Data Figures.

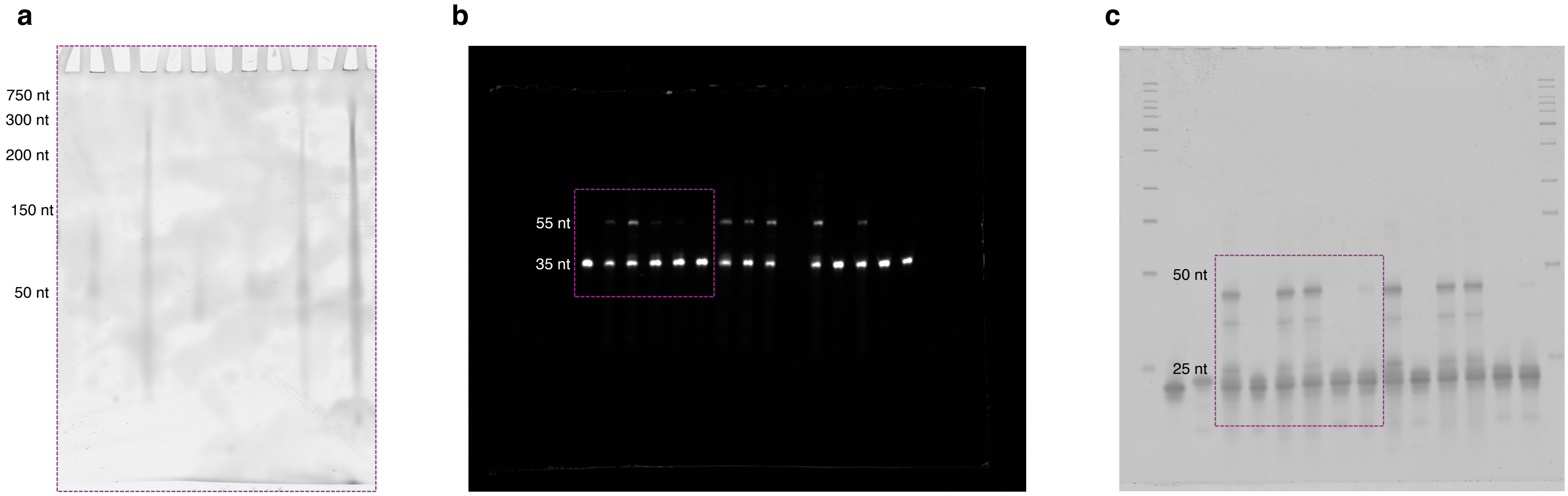

Supplementary Figure 2 | Uncropped polyacrylamide gel electrophoresis blots from Extended Data Figure 3. a, Polyacrylamide gel electrophoresis blot for **Extended Data Figure 3b** showing the relative quantification of nuclear run-on efficiency with various sarkosyl concentrations. Nascent RNA collected after nuclear run-on reaction with either native CTP or click-compatible 3'-(O-Propargyl)-CTP was clicked with Cy5-azide, resolved in denaturing polyacrylamide gel electrophoresis, and imaged for Cy5 fluorescence. **b,** Polyacrylamide gel electrophoresis blot for **Extended Data Figure 3c** shows the effect of different ratios of CuAAC accelerating ligand BTTAA in CuAAC efficiency. RNA-propargyl was clicked with azide-DNA containing Cy5 in the presence of various ratios of BTTAA:$CuSO_4$, resolved in denaturing polyacrylamide gel electrophoresis and imaged for Cy5 fluorescence. **c,** Polyacrylamide gel electrophoresis blot for **Extended Data Figure 3e** shows the effect of the denaturing agent's presence in CuAAC efficiency. The CuAAC reaction was resolved in a denaturing polyacrylamide gel electrophoresis and quantified by staining with SYBR Gold and measuring fluorescence. In panels **a-c**, the purple dotted line represents the gel region shown in Extended Data Figures.

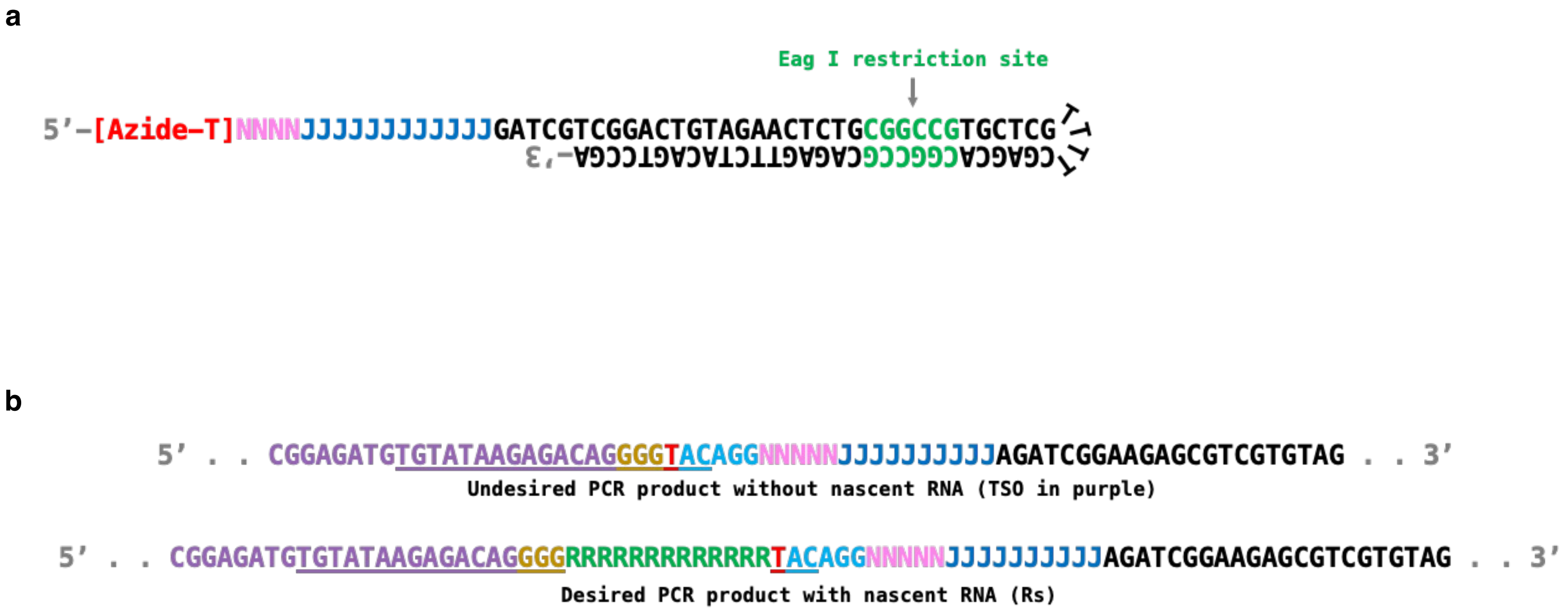

Supplementary Figure 3 | Features of single-cell barcoded DNA adaptors used in scGRO-seq experiments. a, The 86 nt 5'-AzScBc DNA makes a hairpin structure, creating an Eag I restriction site. The 3' end of the adaptor also serves as the extension primer during reverse transcription. **b**, The inclusion of template-switching oligo without CuAAC of nascent RNA on the 43 nt 5'-AzScBc DNA with the ACAGG sequence (top cartoon) forms a substrate sequence for DASH. The CuAAC of nascent RNA fails to generate the substrate for DASH (bottom cartoon).

$$\text{Burst size} = 1 + \frac{\textit{Average reads per burst} - 1}{\textit{Capture efficiency}}$$

$$\text{Burst frequency} = \frac{Nbursts \,/\, Nalleles \,/\, \textit{transcription time}}{\textit{minimum}(\textit{burst size} * \textit{capture efficiency},\ 1)}$$

Supplementary Figure 4 | Equations for the burst size and burst frequency estimation.
