# Supplementary material for: Single-cell nascent RNA sequencing unveils coordinated global transcription
Source: Nature. 2024 Jun 5;631(8019):216–23. doi: 10.1038/s41586-024-07517-7 (PMC11222150; doi:10.1038/s41586-024-07517-7)
Supplement: Supplementary file 2 — Reporting Summary [file 41586_2024_7517_MOESM2_ESM.pdf]

nature portfolio

Corresponding author(s): Phillip A. Sharp

Last updated by author(s): Mar 11, 2024

Reporting Summary

Nature Portfolio wishes to improve the reproducibility of the work that we publish. This form provides structure for consistency and transparency in reporting. For further information on Nature Portfolio policies, see our Editorial Policies and the Editorial Policy Checklist.

Statistics

For all statistical analyses, confirm that the following items are present in the figure legend, table legend, main text, or Methods section.

n/a	Confirmed	
☐	☒	The exact sample size (*n*) for each experimental group/condition, given as a discrete number and unit of measurement
☐	☒	A statement on whether measurements were taken from distinct samples or whether the same sample was measured repeatedly
☐	☒	The statistical test(s) used AND whether they are one- or two-sided<br>*Only common tests should be described solely by name; describe more complex techniques in the Methods section.*
☐	☒	A description of all covariates tested
☐	☒	A description of any assumptions or corrections, such as tests of normality and adjustment for multiple comparisons
☐	☒	A full description of the statistical parameters including central tendency (e.g. means) or other basic estimates (e.g. regression coefficient) AND variation (e.g. standard deviation) or associated estimates of uncertainty (e.g. confidence intervals)
☐	☒	For null hypothesis testing, the test statistic (e.g. *F*, *t*, *r*) with confidence intervals, effect sizes, degrees of freedom and *P* value noted<br>*Give P values as exact values whenever suitable.*
☒	☐	For Bayesian analysis, information on the choice of priors and Markov chain Monte Carlo settings
☒	☐	For hierarchical and complex designs, identification of the appropriate level for tests and full reporting of outcomes
☐	☒	Estimates of effect sizes (e.g. Cohen's *d*, Pearson's *r*), indicating how they were calculated

Our web collection on statistics for biologists contains articles on many of the points above.

Software and code

Policy information about availability of computer code

Data collection	No software except Illumina NExtSeq 5000 and NovaSeq basecalling was used.
Data analysis	The R (v 4.2.2) scripts written in Jupyterlab (v 3.4.3) used for data analyses are publicly available on GitHub (https://github.com/jaymahat/scGROseq). We additionally used Python (v 3.6.4), cutadapt (v 1.16), bamtools (v 2.5.1), samtools (v 1.10), bedtools (v2.29.2), bowtie2 (v 2.3.5.1), flexbar (v 3.5), fastqc (v 0.11.5), DESeq2 (v 1.38.0), Seurat (v 4.3.0), clusterProfiler (v 4.6.0), IGV (v 2.13.0), and GSEA (v 4.3.3). Transcription unit calling was performed using groHMM (https://github.com/dankoc/groHMM)). Enhancers were called using dREG-HD (https://github.com/Danko-Lab/dREG.HD).

For manuscripts utilizing custom algorithms or software that are central to the research but not yet described in published literature, software must be made available to editors and reviewers. We strongly encourage code deposition in a community repository (e.g. GitHub). See the Nature Portfolio guidelines for submitting code & software for further information.

Data

Policy information about availability of data

All manuscripts must include a data availability statement. This statement should provide the following information, where applicable:

- Accession codes, unique identifiers, or web links for publicly available datasets
- A description of any restrictions on data availability
- For clinical datasets or third party data, please ensure that the statement adheres to our policy

The raw and processed data generated in this study are deposited in Gene Expression Omnibus under accession number GSE242176. The published datasets analyzed for this study were obtained from the GEO repository (GSE169044, GSM2360934, GSE169044, GSM1082347, GSM318444, GSM281695, GSM1082344, GSM594579, GSM1082340, GSM1082341, GSM1082342), Supplementary Table S1 of a published manuscript (https://doi.org/10.1016/j.cell.2018.05.035) and reprocessed, and 41586_2018_836_MOESM5_ESM.xlsx file of a published manuscript (https://doi.org/10.1038/s41586-018-0836-1).

Research involving human participants, their data, or biological material

Policy information about studies with human participants or human data. See also policy information about sex, gender (identity/presentation), and sexual orientation and race, ethnicity and racism.

Reporting on sex and gender	N/A
Reporting on race, ethnicity, or other socially relevant groupings	N/A
Population characteristics	N/A
Recruitment	N/A
Ethics oversight	N/A

Note that full information on the approval of the study protocol must also be provided in the manuscript.

Field-specific reporting

Please select the one below that is the best fit for your research. If you are not sure, read the appropriate sections before making your selection.

☒ Life sciences ☐ Behavioural & social sciences ☐ Ecological, evolutionary & environmental sciences

For a reference copy of the document with all sections, see nature.com/documents/nr-reporting-summary-flat.pdf

Life sciences study design

All studies must disclose on these points even when the disclosure is negative.

Sample size	We performed scGRO-seq on 39 96-well plates and 3,744 cells, of which 36 plates and 2,635 cells passed the threshold.<br><br>No Sample size calculation was performed. We collapsed the scGRO-seq libraries to generate psuedo-bulk and compared against inAGTuC, AGTuC, and PRO-seq library prepared from millions of nuclei. We found robust recapitulation of nascent-RNA profiles generated from 2,635 single cells and deemed that the scGRO-seq sample size is sufficient for the analyses we performed in this manuscript.
Data exclusions	The scGRO-seq batches with r2 of at least 0.6 against at least 60% of all batches were selected for further analysis. Cells were required to contain a minimum of 1,000 UMIs and 750 features for further analysis.
Replication	We performed scGRO-seq on 39 96-well plates and 3,744 cells, of which 36 plates and 2,635 cells passed the threshold.<br><br>The 36 replicates of scGRO-seq libraries were prepared over the span of three years. The robustness of correlation among various batches as presented in Extended Data Fig. 7a demonstrates the reproducibility of scGRO-seq method.<br><br>At least two replicates were prepared for inAGTuC and AGTuC libraries.
Randomization	Mouse embryonic stem cells after run-on with 3'-O-Propargyl NTPs were randomly sorted into 96-well plates. 16 frozen 96-well plates with single mES cell in each well out of 40-60 plates prepared for each experiment were randomly selected for scGRO-seq library preparation.
Blinding	Blinding was not necessary. We performed scGRO-seq library preparation 39 times and the samples were randomly handled by three researches at various stages. The roles assigned in tissue culture of mES cells, harvesting of nuclei, run-on with 3'-O-Propargyl NTPs was random among the three researchers.

Reporting for specific materials, systems and methods

We require information from authors about some types of materials, experimental systems and methods used in many studies. Here, indicate whether each material, system or method listed is relevant to your study. If you are not sure if a list item applies to your research, read the appropriate section before selecting a response.

Materials & experimental systems

n/a	Involved in the study
☒	☐ Antibodies
☐	☒ Eukaryotic cell lines
☒	☐ Palaeontology and archaeology
☒	☐ Animals and other organisms
☒	☐ Clinical data
☒	☐ Dual use research of concern
☒	☐ Plants

Methods

n/a	Involved in the study
☒	☐ ChIP-seq
☒	☐ Flow cytometry
☒	☐ MRI-based neuroimaging

Eukaryotic cell lines

Policy information about cell lines and Sex and Gender in Research

Cell line source(s)	V6.5 mouse embryonic stem cells (mESCs) was used in this study. It was established by the Jaenisch laboratory (Whitehead Institute, Massachusetts Institute of Technology) from the inner cell mass (ICM) of a 3.5-day-old mouse embryo from a C57BL/6(F) X 129/sv(M) cross.
Authentication	V6.5 mouse embryonic stem cells were authenticated under microscope for tissue culture phenotype, size of nuclei during FACS, and more importantly, the nascent RNA profile compared with previously published nascent RNA profiles from mouse embryonic stem cells.
Mycoplasma contamination	Cell lines were tested for mycoplasma contamination on a regular basis using a PCR-based test and confirmed for mycoplasma-free.
Commonly misidentified lines (See ICLAC register)	N/A

Plants

Seed stocks	*Report on the source of all seed stocks or other plant material used. If applicable, state the seed stock centre and catalogue number. If plant specimens were collected from the field, describe the collection location, date and sampling procedures.*
Novel plant genotypes	*Describe the methods by which all novel plant genotypes were produced. This includes those generated by transgenic approaches, gene editing, chemical/radiation-based mutagenesis and hybridization. For transgenic lines, describe the transformation method, the number of independent lines analyzed and the generation upon which experiments were performed. For gene-edited lines, describe the editor used, the endogenous sequence targeted for editing, the targeting guide RNA sequence (if applicable) and how the editor was applied.*
Authentication	*Describe any authentication procedures for each seed stock used or novel genotype generated. Describe any experiments used to assess the effect of a mutation and, where applicable, how potential secondary effects (e.g. second site T-DNA insertions, mosiacism, off-target gene editing) were examined.*
